# Ganglion Cell Layer Thickness as a Biomarker for Amyotrophic Lateral Sclerosis Functional Outcome: An OCT study

**DOI:** 10.22336/rjo.2025.32

**Published:** 2025

**Authors:** Divya Singh, Somya Singhal, Vikas Kanaujiya, Ankita Ranjan, Vinita Elizabeth Mani, Vimal Kumar Paliwal, Vaibhav Jain, Ankita Aishwarya, Rachna Agarwal

**Affiliations:** 1 Department of Ophthalmology, Sanjay Gandhi Post Graduate Institute of Medical Sciences, Lucknow, India; 2 Department of Neurology, Sanjay Gandhi Post Graduate Institute of Medical Sciences, Lucknow, India

**Keywords:** ALS, RNFL, GCIPL, ALSFRS, structural outcome, functional outcome, AD = Alzheimer's disease, ALS = amyotrophic lateral sclerosis, ALSFRS = ALS functional rating scale, BCVA = best corrected visual acuity, CNS = central nervous system, CMT = central macular thickness, CST = central subfield thickness, DTI = diffusion tensor imaging, ESA = existing segmentation algorithm, EEC = El Escorial criteria, ELM OLM = external or outer limiting membrane, EMG = electromyography, ETDRS = early treatment for diabetic retinopathy study, FEV 1 = forced expiratory volume at 1 sec, FVC = forced vital capacity, FD OCT = Fourier domain optical coherence tomography, FWHM = full width half maximum, GCIPL = ganglion cell inner plexiform layer, HCDR = horizontal cup to disc ratio, HC = healthy controls, ILM = internal limiting membrane, IMR = inner macular ring, IOP = intraocular pressure, IS OS = inner segment outer segment junction, INL = inner nuclear layer, IPL = inner plexiform layer, LMN = lower motor neuron, MND = motor neuron disease, MRI = magnetic resonance imaging, MT = macular thickness, NFL = nerve fibre layer, NDD = neurodegenerative disorder, NCV = nerve conduction velocity, ONH = optic nerve head, OCT = optical coherence tomography, ONL = outer nuclear layer, OPL = outer plexiform layer, OPTN = optineurin, OMR = outer macular ring, PD = Parkinson’s disease, PFN = profilin gene, pRNFL = peripapillary RNFL, RNFL = retinal nerve fibre layer, RPE = retinal pigment epithelium, SD OCT = spectral domain optical coherence tomography, SS OCT = swept source optical coherence tomography, SLD = super luminescent diode, SNR = signal to noise ratio, SPSS = statistical package for social sciences, TBK 1 = TANK binding kinase 1 gene, TD OCT = time domain optical coherence tomography, UMN = upper motor neuron, UCVA = uncorrected visual acuity, VCDR = vertical cup to disc ratio, VEP = visually evoked potential

## Abstract

**Aim:**

This study aims to evaluate various optical coherence tomography (OCT) parameters in patients diagnosed with amyotrophic lateral sclerosis (ALS).

**Methods:**

Assessment of BCVA was done using Snellen charts, and subjective refraction was done to achieve a BCVA for distance and near. Measurement of intraocular pressure (IOP) was done with Goldman applanation tonometry. Stereoscopic fundus examination was performed using a 90D lens to assess the status of the optic nerve and retina, ruling out any ocular pathology. The patients were then subjected to OCT scanning to measure optic nerve head and macular parameters. Optical coherence tomography was performed using CIRRUS™ HD OCT (500-21822) (version 8.0.0.518) (Carl Zeiss Meditec, Dublin, CA, USA). The analyzed area was centered manually, and the absence of segmentation errors was confirmed for each scan.

**Results:**

RE Avg RNFL and LE Avg RNFL showed weak correlations with ALSFRS, indicated by Pearson Correlation coefficients of 0.073 and -0.026, respectively. The p-values (0.637 and 0.86) suggested that these correlations were not statistically significant. RE Avg GCL and LE Avg GCL, on the other hand, exhibited moderate positive correlations with ALSFRS scores, with correlation coefficients of 0.337 (RE) and 0.389 (LE). These correlations were statistically significant, as indicated by p-values of 0.021 and 0.006, respectively, suggesting a substantial association between GCL thickness and ALS functional outcomes.

**Discussion:**

All patients in our study were clinically diagnosed cases of ALS, as per the El Escorial criteria. Age group-wise analysis showed statistically significant thinning overall as well as quadrant-wise RNFL parameters in patients less than 50 years compared to age-matched controls, indicating that the pathological process occurring in larger motor neurons in ALS might also be happening in smaller sensory neurons of the retina, causing thinning, which was not due to age-related process. Although GCIPL thinning was occurring in our cases, though statistically not significant compared to control, the significant positive correlation observed between GCIPL and ALS functional outcome and between RNFL and GCIPL measurements highlighted the fact that though the axonal degeneration in retinal neurons might not be translating to the same extent in ganglion cells in ALS, the subtle thinning of GCIPL correlated strongly with functional disability in patients with ALS, implying better functional scores with higher values of GCIPL parameters.

**Conclusion:**

In summary, GCL measurements in both eyes showed a notable relationship with ALSFRS, whereas RNFL did not appear to correlate significantly.

## Introduction

Neurodegenerative disorders (NDDs) are characterized by selective loss of neurons, which causes distinctive effects on the functional systems and therefore have characteristic clinical presentations. These disorders share several features, including aberrant protein aggregation, a slow and relentless natural history [1], and an insidious onset in which neuropathological changes develop years before clinical presentation [2]. Diagnostic difficulty in the early stages is due to the heterogeneity of their initial presentation. Examples of NDDs include Alzheimer’s disease (AD), Parkinson’s disease (PD), Amyotrophic Lateral Sclerosis (ALS)/Motor neuron disease (MND), Huntington’s disease, and Frontotemporal dementia. Usually, by the time a patient presents in a tertiary set-up, the disease is well advanced, and a significant amount of neuronal tissue has been irreversibly lost.

Post-mortem tissue studies have corroborated this hypothesis, showing loss of tissue thickness in Alzheimer’s disease (AD) [3-5], Parkinson’s disease (PD) [6], and Amyotrophic Lateral Sclerosis (ALS) [7-11].

ALS is a progressive, paralytic disorder characterized by degeneration and loss of motor neurons in the brain (specifically, pyramidal cells in the cortex and the corticospinal tract) and spinal motor neurons, typically with sparing of the extraocular and sphincter muscles [12-14]. The diagnosis of ALS relies on the medical history, physical examination, electrodiagnostic testing (with needle EMG), and neuroimaging. The gold standard for ALS diagnosis is the El Escorial Criteria, as revised in 1998 [15]. These criteria are primarily based on clinical findings, although they also consider the results of electrophysiological studies.

The non-invasiveness, faster speed, reproducibility, affordability and ease of use have enabled OCT to become an increasingly valuable tool in the assessment of structural changes in axons of the retina and optic nerve in other neurodegenerative disorders such as Alzheimer’s, Parkinson’s disease, multiple sclerosis, neuromyelitis optica etc., which parallel the CNS changes detected on radiological investigations that are more time-consuming, invasive and produce radiation hazards [16-24]. Since alterations in specific regions of the central nervous system (CNS) have been well-documented in these disorders, it is reasonable to assume that the retina and optic nerve, which are anatomical and physiological extensions of the CNS, may also exhibit changes corresponding to the neurodegeneration in other areas. Being the only portion of the CNS available for noninvasive imaging and visualization, such pathological changes, if associated, can be quantified objectively using Optical coherence tomography. Hence, OCT may serve as a valuable tool in the hands of clinicians in quantifying and monitoring axonal and neuronal loss that might be associated with disease progression.

This study aimed to evaluate various optical coherence tomography (OCT) parameters in patients diagnosed with amyotrophic lateral sclerosis (ALS). We have estimated OCT RNFL and GCIPL parameters in cases with ALS and aimed to correlate the severity of ALS with OCT findings.

## Methods

This was a prospective, observational, cross-sectional study conducted from October 2022 to June 2024. We did a literature review and did not find any similar study in the North Indian population. We conducted a pilot study involving 11 patients. At a 95% level of significance, the sample size for the central subfield thickness parameter was maximum. The sample size was further calculated as 51.

### Inclusion criteria of participants (with ALS)

In this study, we included patients with a best-corrected visual acuity of 6/18 or better in both eyes, having a refractive error between -3 and +3 diopters. Any grade of cataract that permitted a signal strength of acquisition of 5 or greater was included. Patients with no e/o hypertensive retinopathy, no e/o diabetic retinopathy, no e/o glaucoma, no other retinal pathology, and no other optic nerve pathology were included.

### Exclusion criteria for participants

Patients with other media opacities, any pre-existing ocular disorder, inability to follow instructions, advanced motor dysfunction that prohibits OCT acquisition, and images with incomplete data were excluded. Lifestyle factors such as smoking, tobacco intake, and alcohol were excluded from both groups.

The study protocol was approved by the institutional research committee and further by the Institutional Ethical Committee of our institution. The study protocol adhered to the principles outlined in the Declaration of Helsinki. Each study participant provided written informed consent before enrollment in the study.

Patients were diagnosed with ALS using the revised El Escorial World Federation criteria. A detailed personal and family history was taken to determine the duration of the disease, the presence of any other systemic or ocular illnesses, the use of any other systemic or ocular medications, any visual, associated, and non-associated complaints, and the degree of functional motor disability. Functional limitation was scored using the rating scale (annex).

Assessment of BCVA was performed using Snellen charts, and subjective refraction was conducted to achieve a best-corrected visual acuity (BCVA) for both distance and near vision. Measurement of intraocular pressure (IOP) was performed using Goldman applanation tonometry, which is considered the gold standard for this measurement. An anterior segment examination was performed using slit lamp biomicroscopy. Following the instillation of mydriatic, a Stereoscopic fundus examination was performed using a 90D lens to assess the status of the optic nerve and retina, ruling out any ocular pathology. The patients were then subjected to OCT scanning to measure the optic nerve head and macular parameters.

Optical coherence tomography was performed using CIRRUS™ HD OCT (500-21822) (version 8.0.0.518) (Carl Zeiss Meditec, Dublin, CA, USA). The same ophthalmologist performed all tests under the same conditions, and the OCT scans were obtained using the same machine.

Average RNFL Thickness in 4 quadrants was distributed evenly around the measurement circle (Superior, Temporal, Inferior, and Nasal).

The total volume of the macula, as determined using OCT, was also used.

The GCC was measured in two inner layers of the retina: the IPL (inner plexiform layer) and the GCL (ganglion cell layer), which were segmented using special software provided by Cirrus HD-OCT Model 500 (Carl Zeiss Meditec, Dublin, CA, USA). The measurements were then reviewed by an ophthalmologist and re-centered if necessary.

The analyzed area was centered manually, and the absence of segmentation errors was confirmed for each scan. All measurements are provided in micrometers (µm), according to the calibration provided by the manufacturers, and the total volume is expressed in cubic millimeters (mm^3^).

The CIRRUS Asian RNFL Normative Database was developed utilizing 315 subjects aged 19 to 79. Subjects were grouped into six categories based on their age: 18-29, 30-39, 40-49, 50-59, 60-69, and 70 and older. Among same-age individuals in the normal population, the percentiles applied to each measurement along the Calculation Circle were as follows: The thinnest 1% of measurements fell in the red area. Measurements in red were considered outside normal limits (red ≤ 1%, outside normal limits). The thinnest 5% of measurements fell in the yellow area or below (1% < green ≤ 95%). The thickest 5% of measurements fell in the white area (white > 95%). Data was analyzed using the Statistical Package for the Social Sciences (SPSS) software version 26.0.

## Results

Fifty-five patients with ALS and 50 healthy controls were included in our study. Of the 55 patients with ALS who were enrolled in the examination, three patients were unable to complete the examination due to positioning difficulties and an inability to follow instructions. Additionally, three patients who underwent OCT examinations had scans that could not be analyzed due to poor scan quality. Hence, 49 patients were scanned. Both eyes of each patient were scanned. A total of 44 right eyes and 48 left eyes were taken. This was because if one eye did not yield a good scan, the other eye was included in our study due to the rare nature of the disease. All the cases of ALS were definite ALS cases following diagnostic confirmation. Cases were also divided based on whether the onset was bulbar or spinal.

Of the 50 healthy controls, 47 patients completed the OCT examination, and three patients were excluded due to an OCT scan suggestive of underlying pathology that could be undiagnosed despite a normal fundus examination.

**Fig. 1:**
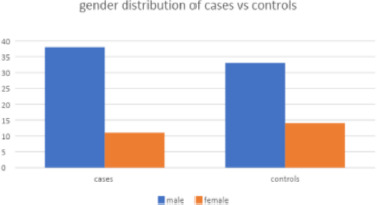
Gender distribution of cases and controls

The gender distribution was analyzed for both cases and controls, as presented in **Table 2** and **Fig. 1**. The case group consisted of 38 males and 11 females. There were 33 males and 14 females in the control group. Using the Chi-square test, it was found that there was no statistically significant difference in the age distribution pattern between the cases and the control group (p = 0.488).

**Table 1:** OCT parameters in the pilot study and sample size calculation

Parameters	Mean	Standard Deviation	d (margin of error)	Sample size
RNFL	86.8	7.5	5	9
GCIPL	77.2	6.6	5	7
CENTRAL SUB FIELD THICKNESS	242.4	18.2	5	50

Similarly, age distribution showed that the mean age amongst cases was 51.71 ± 12.63 years, whereas in the control group, it was 50.49 ± 12.06 years. An independent t-test revealed no significant difference in the mean age between the two groups (p = 0.62) (**Table 2**).

**Table 2:** Demographics

		Case	Controls	p value
Gender	Male	38	33	0.488
	Female	11	14	
Age		51.71 ± 12.63	50.49 ± 12.06	0.62

The mean duration of the disease was 13.21 years.

Comparison between the mean UCVA (logMAR scale) and mean IOP in cases and controls revealed that UCVA in the right eye (RE) in cases was 0.39 ± 0.35. In contrast, in controls, it was 0.14 ± 0.16, which was statistically significant at a p-value of <0.01. Similarly, UCVA in LE for cases was 0.36 ± 0.31, and amongst controls it was 0.13 ± 0.16, which was also statistically significant at a p-value of <0.01.

Comparison of intraocular pressure revealed that the mean IOP in the right eye (RE) was 18.35 ± 3.83 mmHg among cases and 17.51 ± 2.09 mmHg among controls, with no statistically significant difference (p = 0.167). For LE, the mean IOP among cases was 17.88 ± 3.55 mmHg, and for controls, it was 17.66 ± 1.81 mmHg, with no statistically significant difference (p = 0.707) (**Table 3**).

**Table 3:** Comparison of intraocular pressure for RE and LE

	Case	Controls	p value
UCVA RE	0.39 ± 0.35	0.14 ± 0.16	<0.01
UCVA LE	0.36 ± 0.31	0.13 ± 0.16	<0.01
IOP RE	18.35 ± 3.83	17.51 ± 2.09	0.167
IOP LE	17.88 ± 3.55	17.66 ± 1.81	0.707

We categorized the cases and controls into two groups based on age: those under 50 years old and those 50 years old or older.

**Fig. 2:**
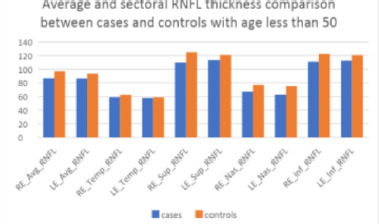
Average and sectoral RNFL thickness comparison between cases and controls with age less than 50

In the age group less than 50 years, the mean RNFL was compared using an independent t-test, which showed that the mean RNFL in the right eye for cases was 86.52 ± 7.95 microns, which was less than that for controls, 96.9 ± 8.76 microns, and this difference was statistically significant with a p-value of <0.001. Similarly, the mean RNFL in the left eye was significantly less in cases (86.45 ± 8.4 microns) than in controls (93.71 ± 7.91 microns; p = 0.007) (**Table 4**) (**Fig. 2**).

**Table 4:** RNFL comparison in cases vs. controls in age less than 50

	Case (age <50)	Control (age < 50)	p-value	Cases (age > 50)	controls (age > 50)	p-value
RE_Avg_RNFL	86.52 ± 7.95	96.9 ± 8.76	<0.001	87.87 ± 10.99	86.23 ± 7.92	0.549
LE_Avg_RNFL	86.45 ± 8.4	93.71 ± 7.91	0.007	87.89 ± 11.57	86.88 ± 7.96	0.713
RE_Temp_RNFL	59 ± 8.34	62.33 ± 9.44	0.232	58.87 ± 9.83	55.92 ± 9.09	0.281
LE_Temp_RNFL	57.75 ± 6.12	58.67 ± 9.27	0.712	58.39 ± 9.98	56.85 ± 10.81	0.587
RE_Sup_RNFL	109.76 ± 12.97	124.86 ± 17.63	0.003	105.3 ± 21.28	105.88 ± 12.9	0.907
LE_Sup_RNFL	113.4 ± 14.67	120.95 ± 15.89	0.122	113.82 ± 21.18	111.19 ± 11.14	0.575
RE_Nas_RNFL	67.19 ± 9.34	77.1 ± 11.47	0.004	64.87 ± 17.34	68.46 ± 9.3	0.363
LE_Nas_RNFL	62.55 ± 9.45	75.1 ± 14.17	0.002	65.93 ± 11.8	66.23 ± 8.47	0.915
RE_Inf_RNFL	110.9 ± 16.38	122.62 ± 16.14	0.025	113.22 ± 29.07	113.5 ± 15.6	0.966
LE_Inf_RNFL	112.3 ± 16.18	120.62 ± 17.1	0.118	112.04 ± 20.55	113.92 ± 14.81	0.702

An independent t-test was used. P <0.05 is significant.

Statistically significant differences were also noted in the superior RNFL quadrant in the right eye which amongst cases was 109.76 ± 12.97 and amongst controls was 124.86 ± 17.63 (p = 0.003), nasal RNFL quadrant in the right eye which amongst cases was 67.19 ± 9.34 and controls was 77.1 ± 11.47 microns (p = 0.004), nasal RNFL quadrant in LE for cases was 62.55 ± 9.45 and controls was 75.1 ± 14.17 microns (p = 0.002) and inferior RNFL quadrant for RE in cases was 110.9 ± 16.38 microns and controls was 122.62 ± 16.14 microns (p = 0.025) (**Table 4**).

Applying the independent t-test for RNFL parameters to an age group of more than 50 years showed that there was no statistically significant difference in average RNFL of the right eye of cases 87.87 ± 10.99 microns and controls 86.23 ± 7.92 microns (p = 0.549) or average RNFL of the left eye of cases 87.89 ± 11.57 microns and controls 86.88 ± 7.96 microns (p = 0.713).

Similarly, no quadrant-specific difference was noted in either the right or left eye of cases and controls in the age group above 50 years (**Table 4**).

For the RNFL comparison, we re-examined GCIPL parameters in the cases and control groups, categorizing them by age into two groups: those under 50 years and those over 50 years, using an independent t-test.

**Fig. 3:**
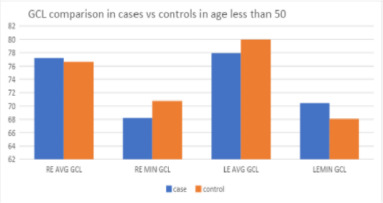
GCL comparison in cases v/s controls, age < 50

In the age group of less than 50 years, the average GCL in the right eye was higher for cases (77 ± 7.41 microns) than for controls (76.61 ± 12.93 microns), but this difference was not statistically significant (p = 0.86) (**Table 5**) (**Fig. 3**).

**Table 5:** GCL comparison in cases vs. controls in age less than 50

		Case	Controls	p value
Age: less than 50 years	RE_Avg_GCL	77 ± 7.41	76.61 ± 12.93	0.86
	RE_Min_GCL	68.19 ± 17.19	70.78 ± 14.46	0.62
	LE_Avg_GCL	77.95 ± 9.70	79.95 ± 7.91	0.47
	LE_Min_GCL	70.43 ± 18.92	68.05 ± 17.40	0.67

An independent t-test was used. P<0.05 is significant.

Comparing the left eyes, the average GCIPL thickness was lower in cases, at 77.95 ± 9.70 microns, than in controls, at 79.95 ± 7.91 microns; however, this difference was not statistically significant (p = 0.47).

In the age group of more than 50 years, there was a minimal difference between the average GCIPL in the right eyes for cases (79.15 ± 7.32 microns) and controls (79.5 ± 5.07 microns) (p = 0.84).

**Fig. 4:**
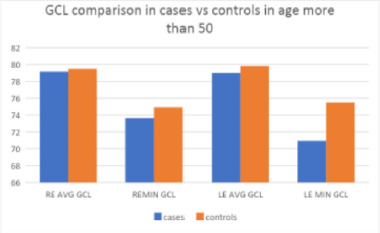
GCL comparison in cases v/s controls, age > 50

Similarly, for the left eyes in the same group, there was a minimal difference between the average GCIPL for cases (79 ± 7.57 microns) and controls (79.81 ± 5.543 microns; p = 0.65) (**Table 6**) (**Fig. 4**).

**Table 6:** GCL comparison in cases vs. controls in age more than 50

Age more than or equal to 50	RE_Avg_GCL	79.15 ± 7.32	79.5 ± 5.07	0.84
	RE_Min_GCL	73.62 ± 10.0	74.92 ± 6.657	0.58
	LE_Avg_GCL	79 ± 7.57	79.81 ± 5.543	0.65
	LE_Min_GCL	70.93 ± 12.9	75.5 ± 7.033	0.11

An independent t-test was used. P<0.05 is significant.

Using Pearson correlation, we compared RNFL parameters and GCIPL parameters amongst cases with their scores on ALSFRS.

Correlation **Table 7** examines the relationship between average retinal nerve fibre layer (RNFL) and ganglion cell layer (GCL) measurements for both the right eye (RE) and left eye (LE), as well as the ALS Functional Rating Scale (ALSFRS) scores.

**Table 7:** Correlations between RNFL and GCIPL and ALSFRS

	ALSFRS score	
	Pearson Correlation	p value
RE_Avg_RNFL	0.073	0.637
LE_Avg_RNFL	-0.026	0.86
RE Avg_GCL	0.337	0.021
LE_Avg_GCL	0.389	0.006

RE Avg RNFL and LE Avg RNFL showed weak correlations with ALSFRS, indicated by Pearson Correlation coefficients of 0.073 and -0.026, respectively. The p-values (0.637 and 0.86) suggested that these correlations were not statistically significant.

RE Avg GCL and LE Avg GCL, on the other hand, exhibited moderate positive correlations with ALSFRS scores, with correlation coefficients of 0.337 (RE) and 0.389 (LE). These correlations were statistically significant, as indicated by p-values of 0.021 and 0.006, respectively, suggesting a substantial association between GCL thickness and ALS functional outcomes.

In summary, GCL measurements in both eyes showed a notable relationship with ALSFRS, whereas RNFL did not appear to correlate significantly.

## Discussion

ALS is characterized by the loss and degradation of spinal motor neurons as well as brain motor neurons, typically leaving the extraocular and sphincter muscles intact. During our study of this progressive paralytic disorder, we found no significant difference in the gender distribution of cases versus controls (Chi-square test, p-value = 0.488). There was no significant difference in the age group distribution of cases vs. controls (independent t-test p-value = 0.62). This ensures the comparability between the cases and control groups. In a 2022 study by Mohanty et al., cases were divided into two groups: those with a short duration (<24 months) and those with a long duration (>24 months). The study reported a mean duration of 29.6 months ± 14.5 months, ranging from 10 to 59 months, with a median of 26 months.

However, in our study, the mean duration of disease in cases was 13.21 ± 9.73 months, ranging from 3 months to 36 months, with a median duration of 9 months. Duration of symptoms is usually self-reported and may be subject to recall bias. Also, some patients may report weakness of limbs earlier than dysarthria and dysphagia, or vice versa. Patients coming to SGPGI are usually referred from other centers and cities across Uttar Pradesh, Bihar, and Madhya Pradesh; therefore, the time of referral is a crucial factor in determining disease duration. Those presenting late to the OPD may have a well-advanced disease. This may preclude such patients from undergoing ophthalmic examination due to motor difficulties.

All our patients were diagnostically confirmed definite ALS cases, unlike a few previous studies that compared possible, probable, and definite ALS cases with the control group. The comparisons in our study were drawn between definite ALS cases and the age-matched control group (p value 0.62) (**Table 8**).

**Table 8:** RNFL average in age less than 50 years

	cases	controls	
RE_Avg_RNFL	86.52 ± 7.95	96.9 ± 8.76	<0.001
LE_Avg_RNFL	86.45 ± 8.4	93.71 ± 7.91	0.007

We observed that in our study, average RNFL values were lower in both the right and left eyes of patients compared to the control group, and that this difference was statistically significant for cases with ages less than 50 years.

Our data supported the hypothesis that ALS patients without ophthalmic disease exhibited retinal thinning, which was more pronounced in the younger age group (less than 50 years), when age-related thinning was less likely to occur. This RNFL thinning in ALS patients compared to controls who were younger than 50 years of age might have been independent of age-related decline in RNFL and might have been suggestive of sensory involvement due to the underlying pathologic pathways in ALS as there was a definite correlation between structural and functional involvement in motor pathways which were more highlighted in younger age group, however with advancing age, the age-related degenerative processes take equal precedence such that this retinal degeneration due to ALS might become masked.

A comparison of GCL between ALS cases and controls did not show statistically significant differences in either age group, those under 50 years or those over 50 years.

To our surprise, though there was definite RNFL thinning in patients less than 50 years of age, using correlational analysis showed that the correlation between RE average RNFL and LE average RNFL with ALSFRS was not statistically significant with Pearson Correlation coefficients of 0.073 (p = 0.637) and -0.026 (p = 0.86) respectively amongst cases. This result was similar to that of Mukherjee et al.

On the other hand, although the independent t-test indicated no significant differences in GCIPL measures between cases and the control group, amongst cases, RE Avg GCL and LE Avg GCL exhibited moderate positive correlations with ALSFRS scores, with correlation coefficients of 0.337 (RE) and 0.389 (LE), respectively. These correlations were statistically significant, as indicated by p-values of 0.021 and 0.006, respectively, suggesting a substantial association between GCL thickness and ALS functional outcomes.

Considering the rare nature of the disease, previous studies done in the West have had fewer patients compared to our research. We have included an age and sex-matched control group that underwent a detailed clinical examination similar to that of the patients with ALS.

Our study excluded the presence of ophthalmic disease both by history and detailed clinical examination by experienced ophthalmologists, unlike the studies done by Roth et al., Ringelstein et al., and Hubers et al.

There were several limitations to our study. None of the patients in our study group reported visual complaints. Motor weakness in ALS patients poses a challenge in conducting a detailed ophthalmic examination. Patients are easily fatigued and may not be able to undergo the whole duration of ophthalmic evaluation which involves clinical refraction, testing for color vision and contrast sensitivity, slit lamp examination before and after pupillary dilatation and then subjecting the patient to OCT evaluation.

Advanced disease may render the patient with severe motor limitations, dyspnea, and excessive salivation, making it impossible for them to undergo such an examination. Consequently, it becomes quite challenging to incorporate ophthalmic examination as part of routine medical care for this group of patients.

## Conclusion

Since our study was cross-sectional, our observations at a single point in time may not accurately correlate with disease severity and progression. Frequent follow-ups may enable us to take repeated measurements of retinal parameters with OCT, and baseline parameters may then be available for subsequent comparisons. This may be more informative in highlighting the correlation between the rate of change in these parameters and disease progression and severity, thereby substantiating their use as biomarkers for ALS.
